# LncRNA KIAA0087 suppresses the progression of osteosarcoma by mediating the SOCS1/JAK2/STAT3 signaling pathway

**DOI:** 10.1038/s12276-023-00972-8

**Published:** 2023-04-03

**Authors:** Haoli Gong, Ye Tao, Sheng Xiao, Xin Li, Ke Fang, Jie Wen, Pan He, Ming Zeng

**Affiliations:** 1 Department of Orthopedics, Hunan Provincial People’s Hospital (The First Affiliated Hospital of Hunan Normal University), Changsha, 410005 Hunan Province P. R. China; 2 Department of Radiology, The Third Xiangya Hospital, Central South University, Changsha, 410013 Hunan Province P. R. China

**Keywords:** Sarcoma, Sarcoma

## Abstract

Long noncoding RNAs (lncRNAs), widely expressed in mammalian cells, play pivotal roles in osteosarcoma (OS) progression. Nevertheless, the detailed molecular mechanisms of lncRNA KIAA0087 in OS remain obscure. Here, the roles of KIAA0087 in OS tumorigenesis were investigated. KIAA0087 and miR-411-3p levels were detected by RT-qPCR. Malignant properties were assessed by CCK-8, colony formation, flow cytometry, wound healing, and transwell assays. SOCS1, EMT, and JAK2/STAT3 pathway-related protein levels were measured by western blotting. Direct binding between miR-411-3p and KIAA0087/SOCS1 was validated by a dual-luciferase reporter, RIP, and FISH assays. In vivo growth and lung metastasis were evaluated in nude mice. The expression levels of SOCS1, Ki-67, E-cadherin, and N-cadherin in tumor tissues were measured by immunohistochemical staining. Downregulation of KIAA0087 and SOCS1 and upregulation of miR-411-3p were found in OS tissues and cells. Low expression of KIAA0087 was associated with a poor survival rate. Forced expression of KIAA0087 or miR-411-3p inhibition repressed the growth, migration, invasion, EMT, and activation of the JAK2/STAT3 pathway and triggered apoptosis of OS cells. However, the opposite results were found with KIAA0087 knockdown or miR-411-3p overexpression. Mechanistic experiments indicated that KIAA0087 enhanced SOCS1 expression to inactivate the JAK2/STAT3 pathway by sponging miR-411-3p. Rescue experiments revealed that the antitumor effects of KIAA0087 overexpression or miR-411-3p suppression were counteracted by miR-411-3p mimics or SOCS1 inhibition, respectively. Finally, in vivo tumor growth and lung metastasis were inhibited in KIAA0087-overexpressing or miR-411-3p-inhibited OS cells. In summary, the downregulation of KIAA0087 promotes the growth, metastasis, and EMT of OS by targeting the miR-411-3p-mediated SOCS1/JAK2/STAT3 pathway.

## Cancer: How one long non-coding RNA suppresses bone tumors

A detailed understanding of how one particular non-coding regulatory RNA molecule suppresses the growth of bone tumors could lead to new treatments for osteosarcoma, the most common form of bone tumor. Ming Zeng of Hunan Provincial People’s Hospital in Changsha, China, and colleagues showed that elevated activity of a long non-coding RNA called KIAA0087 reduces the proliferation, migration, and invasiveness of osteosarcoma tumors, both in cell culture experiments and in mice. The authors detailed how KIAA0087 binds to a smaller regulatory RNA and prevents it from inhibiting the expression of the protein it regulates. That protein is then expressed and is free to inactivate a signaling pathway that normally enhances tumor growth. These mechanistic insights offer several potential points of intervention for drug developers to create new targeted anti-cancer agents.

## Introduction

Osteosarcoma (OS) is a bone malignancy that most commonly occurs in children and adolescents^1^. Globally, 4–5 per million patients are diagnosed with OS annually^2^. The current treatments for OS are surgery in combination with chemotherapy or radiotherapy as well as administration of novel antitumor agents or molecularly targeted therapies^3^. Nevertheless, the 5-year survival rate for OS patients is between 40% and 70%^2,4^, which can decline to only 19% when metastasis occurs^5^. With increasing research on the pathogenesis of OS, much attention has been given to epithelial-mesenchymal transition (EMT). EMT is a transformation of polarized epithelial cells to mesenchymal cells^6^. EMT drives the tumorigenesis and progression of OS by enhancing the metastatic ability of cancer cells^7^. Due to the promotive roles of EMT in OS progression, inhibition of EMT has been considered a promising therapeutic strategy.

Long noncoding RNA (lncRNA), a novel kind of noncoding RNA, is composed of more than 200 nucleotides without coding potential. Abnormal expression of certain lncRNAs is significantly associated with the malignant phenotypes of tumor cells^8^. A previous study revealed that lncRNA KIAA0087 levels were reduced in endometrial carcinoma and participated in endometrial carcinogenesis^9^. To date, the expression and functional roles of KIAA0087 in OS have not been revealed. A new theory that lncRNAs may counteract the biological activities of microRNAs (miRNAs) by sponging them has been widely accepted^10^. MiRNAs are another kind of noncoding RNA composed of 17–25 nucleotides; these RNAs repress the translation of target genes by complementary base pairing, and this function of miRNAs has been shown to be involved in the pathogenesis of tumors^11^. Many differentially expressed miRNAs have been documented to regulate the malignancy of OS cells^12,13^. Recently, one study reported the upregulation of miR-411-3p in OS cell lines^14^. However, the functional roles of dysregulated miR-411-3p in OS are unclear. Importantly, bioinformatics analysis (based on the LncBase database) shows that KIAA0087 can bind to miR-411-3p, although no studies have reported an interaction between them. Therefore, KIAA0087 might affect OS progression by regulating miR-411-3p expression.

The suppressor of cytokine signaling 1 (SOCS1) is a member of the SOCS family. SOCS1 is considered a suppressor or promotor of different cancers^15^. A reduced SOCS1 level mediated by promoter methylation has been shown to facilitate EMT and the development of acute myeloid leukemia cells by activating the JAK2/STAT3 signaling pathway^16^. In contrast, another study demonstrated that SOCS1 deficiency slowed tumor development by increasing antitumor inflammation^17^. To date, only one study has indicated that SOCS1 methylation confers malignant properties to OS cells^18^. Notably, based on the StarBase database, SOCS1 is predicted to be a target of miR-411-3p. Therefore, the miR-411-3p/SOCS1 axis might participate in KIAA0087-regulated OS progression.

In this study, we found decreased KIAA0087 and elevated miR-411-3p expression in OS tissues and cells, which contributed to the proliferation, migration, invasion, and EMT of OS cells. KIAA0087 sponged miR-411-3p to enhance SOCS1 expression and subsequently suppressed JAK2/STAT3 pathway-mediated EMT. Our study suggested the potential of KIAA0087 as a novel target and prognostic indicator for OS.

## Materials and methods

### Clinical samples

Clinical OS tissues and matched normal tissues were collected from 30 OS patients during surgery at Hunan Provincial People’s Hospital (The First Affiliated Hospital of Hunan Normal University). The collected samples were snap-frozen in liquid nitrogen and preserved at −80 °C. None of the patients had previously undergone chemoradiotherapy, and all the patients signed written informed consent. Our experiments were approved by the Ethics Committee of Hunan Provincial People’s Hospital (The First Affiliated Hospital of Hunan Normal University). Table 1 presents the clinicopathological characteristics of these patients.

**Table 1:** Associations between KIAA0087/miR-411-3p expression and clinicopathological characteristics in osteosarcoma patients.

Clinical parameter	Cases (*n*)	Expression level		*P* value	Expression level		*P* value
		KIAA0087^high^	KIAA0087^low^		miR-411-3p^high^	miR-411-3p^low^	
*Age (years)*							
<18 years	20	9	11	0.6999	12	8	0.2451
≥18 years	10	6	4		3	7	
*Sex*							
Male	17	7	10	0.4621	8	9	1.0000
Female	13	8	5		7	6	
*Tumor size (cm)*							
<5 cm	12	9	3	0.0604	5	7	0.7104
≥5 cm	18	6	12		10	8	
*TNM stage*							
I	14	12	2	0.0007	4	10	0.0656
II + III	16	3	13		11	5	
*Distant metastasis*							
Yes	16	7	9	0.7152	12	4	0.0092
NO	14	8	6		3	11	

### Cells and treatment

OS cells (U2OS, Saos-2, MG-63, and HOS) and human osteoblasts (hFOB 1.19) were obtained from the Cell Bank of the Chinese Academy of Science (Shanghai, China). U2OS (MEM, Gibco, Grand Island, NY, USA), Saos-2 (MEM, Gibco), MG-63 (McCOY’s 5A, Gibco), HOS (McCOY’s 5A, Gibco), and hFOB 1.19 (DMEM, Gibco) cells were cultured in an appropriate medium containing 10% fetal bovine serum (FBS, Gibco) and 1% penicillin/streptomycin (Invitrogen, CA, USA) and incubated at 37 °C with 5% CO_2_.

### Lentivirus infection

The lentivirus vector used to overexpress KIAA0087 (OE-KIAA0087), short hairpin RNA used to silence KIAA0087 and SOCS1 (sh-KIAA0087 and sh-SOCS1), their negative controls (OE-NC and sh-NC), miR-411-3p mimics, miR-411-3p inhibitor, and mimics/inhibitor NC were purchased from GeneChem (Shanghai, China). U2OS and Saos-2 cells were infected with the above lentiviruses with 5 μg/mL polybrene (GeneChem).

### Cell counting kit-8 (CCK-8)

The proliferation of OS cells was assessed at various time points (24, 48, and 72 h) with a CCK-8 Kit (Bestbio, Shanghai, China) strictly in accordance with the manufacturer’s instructions. The results were obtained using a multimode reader at 450 nm (Tecan, Männedorf, Switzerland).

### Colony formation assay

The proliferation of OS cells was evaluated by colony formation. After culturing 100 cells in 6-well plates for 2 weeks, the clones were fixed with 4% paraformaldehyde and stained with 0.5% crystal violet solution. Subsequently, the colony number was calculated.

### Cell apoptosis detection

An Annexin V-FITC/PI Apoptosis Detection Kit (Thermo Fisher Scientific) was adopted for apoptosis detection. Briefly, OS cells were stained with Annexin V-FITC (5 μL) and PI Staining Solution (10 μL). After a reaction for 10 min in the dark, 400 μL binding buffer was added, followed by detection by flow cytometry (Becton-Dickinson).

### Wound healing assay

The migration of OS cells was determined by wound healing assay. A scratch was made in confluent OS cells seeded in 24-well plates using a micropipette tip. Then, the cells were permitted to migrate in a serum-free medium for 24 h. The cells were imaged with a microscope (Zeiss, Germany) at 0 and 24 h after scratch treatment.

### Transwell assay

OS cells in 200 μL serum-free medium were added to the upper chamber of a transwell system coated with Matrigel (BD Biosciences, San Jose, CA, USA). The lower chamber was filled with 600 μL medium containing 10% FBS. After invading for 24 h in an incubator, the noninvaded cells in the upper chamber were removed by swabbing, and the invaded cells on the lower surface of the chamber were fixed in 4% paraformaldehyde, stained with 0.5% crystal violet, and photographed under a microscope (Zeiss, Germany).

### Dual-luciferase reporter assay

The interaction between KIAA0087 and miR-411-3p was predicted using the LncBase database (http://carolina.imis.athena-innovation.gr/diana_tools/web/index.php?r=lncbasev2/index-predicted). The direct binding between the SOCS1 3′-UTR and miR-411-3p was predicted using the StarBase database (http://starbase.sysu.edu.cn/index.php). The sequences of wild-type (WT) KIAA0087 and SOCS1 3′-UTR containing miR-411-3p binding sites or the mutant (MUT) sequences were obtained by PCR amplification and subcloned into the psiCHECK2 vector (Promega, Madison, Wisconsin, USA). OS cells were cotransfected with KIAA0087-WT, SOCS1-WT, or SOCS1-MUT vectors with miR-411-3p mimics or inhibitors. Subsequently, the luciferase activity of OS cells was assessed with a Dual-Lucy Assay Kit (Solarbio, Beijing, China) after 48 h.

### RNA immunoprecipitation (RIP) assay

A Magna RIP RNA-Binding Protein Immunoprecipitation Kit (Millipore, Billerica, MA, USA) was used after OS cells were transfected with miR-411-3p mimics or NC mimics. Magnetic beads conjugated to Ago2 (Millipore) or nonspecific IgG antibody (Millipore) were applied for incubation with cell lysates at 4 °C overnight. After treatment with proteinase K, the level of KIAA0087 in the immunoprecipitated RNAs was assessed by qPCR.

### Fluorescence in situ hybridization (FISH) assay

Cy3-labeled KIAA0087 and FITC-labeled miR-411-3p probes were purchased from GenePharma (Shanghai, China). The probe signals were determined with a FISH Probe Kit from GenePharma. Nuclei were stained with DAPI (Solarbio). The co-localization of KIAA0087 and miR-411-3p in OS cells was observed under a fluorescence microscope (Zeiss, Germany).

### Animal studies

The animal experiments were approved by the Animal Ethics Committee of Hunan Provincial People’s Hospital (The First Affiliated Hospital of Hunan Normal University, IRB number: 20210021). Six-week-old male BALB/C nude mice were purchased from Shanghai SLAC Laboratory Animal Center (Shanghai, China). For the tumorigenesis experiment, mice have subcutaneously injected with 1 × 10^7^ U2OS or Saos-2 cells stably infected with lentiviruses carrying KIAA0087 or miR-411-3p inhibitor (*n* = 6 per group). All mice were sacrificed 30 days after injection, and the xenograft tumors were collected and weighed.

For the metastasis experiment, the mice were injected via the tail vein with 5 × 10^6^ U2OS or Saos-2 cells stably infected with lentiviruses carrying KIAA0087 or miR-411-3p inhibitor. Four weeks later, all mice were sacrificed, and lung metastasis was evaluated. The obtained lung tissues were fixed in 10% formalin for subsequent experiments.

### Hematoxylin and eosin (H&E) staining

For lung metastasis analysis, the lung tissues of nude mice were subjected to paraffin embedding and cut into 5 μm-thick serial sections, stained with H&E solution (Abcam), and photographed under a light microscope (Zeiss, Germany).

### Immunohistochemical staining

The prepared paraffin sections were dewaxed, rehydrated, and incubated in 3% H_2_O_2_. Then, antigen retrieval was performed by heating in citrate buffer. The sections were probed with primary antibodies against SOCS1 (1:200, ab9870, Abcam), Ki-67 (1:500, ab15580, Abcam), E-cadherin (1:400, #3195, Cell Signaling Technology, Danvers, MA, USA), and N-cadherin (1:100, #13116, Cell Signaling Technology) overnight at 4 °C. Thereafter, the biotinylated secondary antibody was applied, followed by visualization with 3,3′-diaminobenzidine (DAB).

### Western blot analysis

Protein isolation from OS cells was carried out using RIPA buffer (Beyotime, Shanghai, China) containing a proteinase inhibitor cocktail. Total protein was quantified with the BCA Protein Assay Kit (Beyotime); proteins were then separated by SDS-PAGE and transferred onto polyvinylidene fluoride membranes (Millipore). The primary antibodies for immunoblotting were as follows: E-cadherin (1:1000, #3195, Cell Signaling Technology), N-cadherin (1:2000, #13116, Cell Signaling Technology), vimentin (1:1500, #5741, Cell Signaling Technology), MMP-2 (1:1000, #40994, Cell Signaling Technology), slug (1:1000, #9585, Cell Signaling Technology), SOCS1 (1:2000, ab62584, Abcam), p-JAK2 (Tyr1007, 1:1000, #4406, Cell Signaling Technology), JAK2 (1:1500, #3230, Cell Signaling Technology), p-STAT3 (Tyr705, 1:1000, ab267373, Abcam), STAT3 (1:1000, ab68153, Abcam), bax (1:2000, ab182733, Abcam), bcl-2 (1:2000, ab182858, Abcam), cleaved caspase-3 (1:500, ab2302, Abcam), Notch1 (1:1000, ab52627, Abcam), Hes1 (1:1000, ab71559, Abcam), β-catenin (1:1000, ab223075, Abcam), c-Myc (1:1000, #13987, Cell Signaling Technology), p-PI3K (Tyr607, 1:1000, ab182651, Abcam), PI3K (1:1000, ab191606, Abcam), p-AKT (Ser473, 1:2000, #4060, Cell Signaling Technology), AKT (1:1000, #4691, Cell Signaling Technology), and GAPDH (1:2000, ab9485, Abcam). These antibodies were applied at 4 °C overnight. After incubation with the secondary antibody, the protein bands were detected using ECL substrate (Thermo Fisher Scientific).

### Real-time quantitative PCR (RT-qPCR)

Total RNA was isolated using the TRIzol RNA Extraction Kit (Engreen Biosystem, Birkenhead, Auckland, New Zealand). Reverse transcription was carried out using the PrimeScript RT Reagent Kit gDNA Eraser (Takara, Dalian, China). The relative expression levels of KIAA0087, SOCS1, RHOB, XRN1, miR-411-3p, miR-30e-5p, miR-135b-5p, miR-6807-3p, and miR-488-3p were determined by qPCR using the SYBR Premix Ex Taq II Kit (TaKaRa). GAPDH and U6 small nuclear RNA were used as internal references for mRNA and miRNA, respectively. The relative quantification of gene expression was determined using the 2^−ΔΔCt^ method.

### Statistical analysis

Data from three biological replicates were analyzed with GraphPad Prism 6.0 and are presented as the mean ± standard deviation (SD). Student’s *t*-test was performed when there were two experimental groups, while a one-way analysis of variance (ANOVA) followed by Tukey’s post hoc test was performed for multiple group comparisons. The overall survival curve of OS patients was analyzed with the Kaplan‒Meier method. Spearman correlation analysis was used to study the relationship between KIAA0087, miR-411-3p, and SOCS1. The correlation between KIAA0087 and miR-411-3p expression and the clinicopathological characteristics of OS patients was assessed with the chi-square test. A *P* value less than 0.05 was considered significant.

## Results

### Downregulation of KIAA0087 indicates a poor prognosis and activates the JAK2/STAT3 pathway in OS

**Fig. 1:**
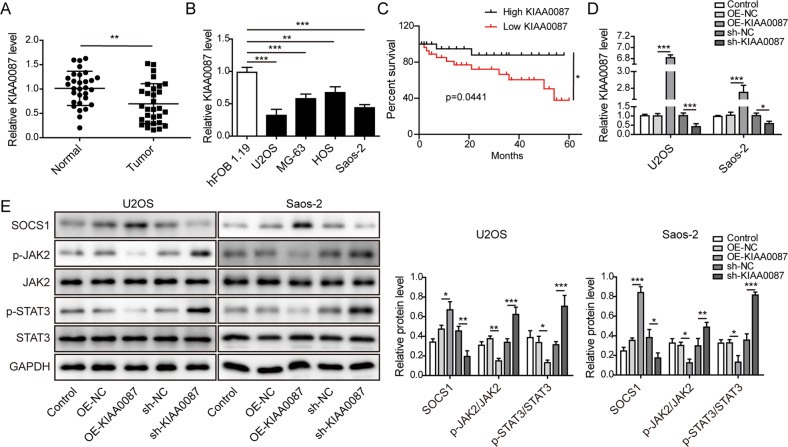
Aberrant low expression of KIAA0087 in OS is associated with a poor prognosis. **a** RT-qPCR analysis was used to evaluate KIAA0087 levels in clinically normal and OS samples (*n* = 30). **b** RT-qPCR analysis was used to evaluate KIAA0087 levels in hFOB 1.19 and different OS cells (U2OS, Saos-2, MG-63, and HOS). **c** The survival rate of OS patients with high or low expression of KIAA0087. **d** The transfection efficiency of overexpression or silencing of KIAA0087 was detected by RT-qPCR. **e** Western blot analysis of the protein levels of SOCS1, p-JAK2, JAK2, p-STAT3, and STAT3. *p < 0.05, **p < 0.01, and ***p < 0.001.

KIAA0087 is a novel lncRNA, and its biological functions in OS remain unclear. We first investigated the expression level of KIAA0087 in OS tissues and cells using RT-qPCR. Significant downregulation of KIAA0087 was found in OS tissues (Fig. 1a), and a series of OS cells (Fig. 1b) compared to the corresponding control groups. Moreover, the survival rate was decreased in patients with lower expression of KIAA0087 than in those with high expression of KIAA0087 (Fig. 1c). To further explore the functional roles of KIAA0087 in EMT, we evaluated the effects of KIAA0087 on EMT-related signaling pathways (JAK2/STAT3, Notch1/Hes1, and Wnt/β-catenin). KIAA0087 was overexpressed or silenced in U2OS and Saos-2 cells (Fig. 1d). The phosphorylation levels of JAK2 and STAT3 were reduced, while the SOCS1 level was enhanced in U2OS and Saos-2 cells after forced expression of KIAA0087. However, the opposite results were found with the loss of KIAA0087 (Fig. 1e). As presented in Supplementary Fig. 1a, b, the expression levels of Notch1, Hes1, β-catenin, and c-Myc in OS cells were not changed after overexpression or knockdown of KIAA0087. Taken together, KIAA0087 was expressed at low levels in OS, which was correlated with a poor prognosis and dysregulation of the JAK2/STAT3 pathway.

### KIAA0087 affects the proliferation and apoptosis of OS cells

**Fig. 2:**
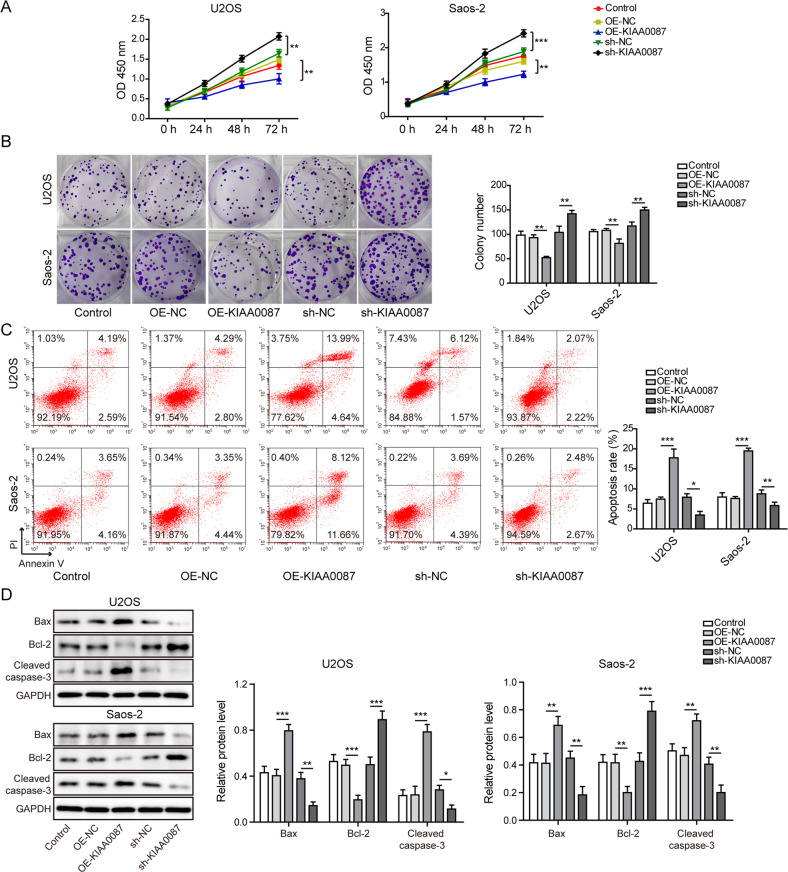
Dysregulation of KIAA0087 affects the proliferation and apoptosis of OS cells. **a** CCK-8 assay determined the proliferation of OS cells. **b** Colony formation assay for evaluating the growth of OS cells. **c** Flow cytometry for testing the apoptotic rate of OS cells. **d** The protein levels of Bax, bcl-2, and cleaved caspase-3 in OS cells were determined by western blotting. *p < 0.05, **p < 0.01, and ***p < 0.001.

Further functional assays indicated that forced expression of KIAA0087 strikingly repressed the growth of both U2OS and Saos-2 cells, while the opposite results were found with the knockdown of KIAA0087 (Fig. 2a, b). In addition, the apoptosis of OS cells was induced by KIAA0087 overexpression but inhibited by its depletion (Fig. 2c). As assessed by western blotting, overexpression of KIAA0087 resulted in upregulation of Bax and cleaved caspase-3 and downregulation of bcl-2 in OS cells, whereas KIAA0087 deficiency led to the opposite results (Fig. 2d). These findings suggested that KIAA0087 repressed the proliferation and induced the apoptosis of OS cells.

### KIAA0087 suppresses the migration, invasion, and EMT of OS cells

**Fig. 3:**
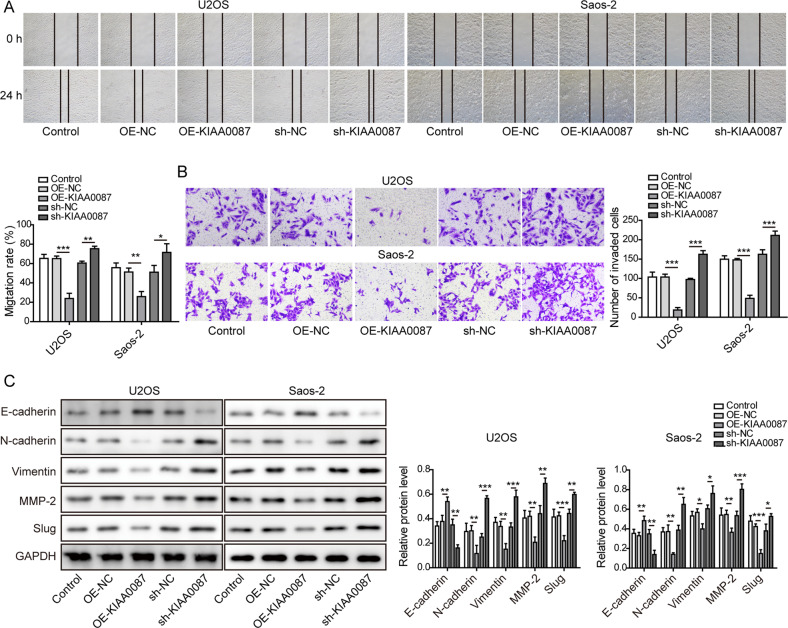
Effect of KIAA0087 on the migration, invasion, and EMT of OS cells. **a** Wound healing assay for detecting the migration of OS cells. **b** Transwell assay for assessing the invasion of OS cells. **c** The protein levels of E-cadherin, N-cadherin, vimentin, MMP-2, and slug were assessed by western blotting. *p < 0.05, **p < 0.01, and ***p < 0.001.

The migration and invasion of OS cells were remarkably suppressed in KIAA0087-overexpressing OS cells but enhanced in KIAA0087-silenced cells (Fig. 3a, b). Thus, we speculated that the EMT process might be regulated by KIAA0087. As detected by western blotting, KIAA0087 overexpression increased E-cadherin expression but decreased N-cadherin, vimentin, MMP-2, and slug expression in OS cells, suggesting the inhibition of EMT. However, KIAA0087 depletion exhibited the opposite effects (Fig. 3c). Collectively, the above findings suggested that the abnormal downregulation of KIAA0087 endowed OS cells with migration, invasion, and EMT abilities.

### KIAA0087 directly interacts with miR-411-3p in OS cells

**Fig. 4:**
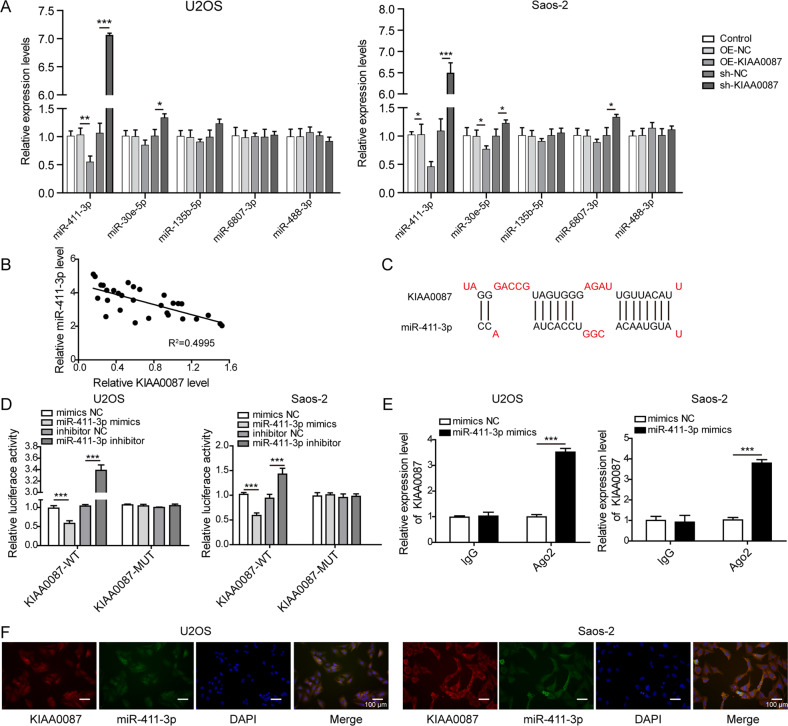
KIAA0087 directly binds to miR-411-3p in OS cells. **a** RT-qPCR detected miR-411-3p, miR-30e-5p, miR-135b-5p, miR-6807-3p, and miR-488-3p expression after overexpression or inhibition of KIAA0087 in OS cells. **b** The correlation between KIAA0087 and miR-411-3p expression in the clinical OS samples was evaluated (*n* = 30). **c** Bioinformatic analysis of the specific binding regions between KIAA0087 and miR-411-3p. **d**, **e** The direct binding of KIAA0087 to miR-411-3p was validated by dual-luciferase reporter assay (**d**) and RIP assay (**e**). **f** The cytoplasmic colocalization of KIAA0087 and miR-411-3p in OS cells was observed by FISH. *p < 0.05, **p < 0.01, and ***p < 0.001.

As predicted by bioinformatics analysis, five target miRNAs (miR-411-3p, miR-30e-5p, miR-135b-5p, miR-6807-3p, and miR-488-3p) of KIAA0087 were screened according to prediction ranking and research status. Furthermore, RT-qPCR results showed that the change in miR-411-3p level was apparently most pronounced among the above five miRNAs after overexpression or knockdown of KIAA0087 in OS cells (Fig. 4a). Furthermore, miR-411-3p was negatively correlated with KIAA0087 levels in OS tissues (Fig. 4b). The LncBase database predicted that miR-411-3p was a potential target of KIAA0087 (Fig. 4c). The relative luciferase activity of the KIAA0087-WT group was dramatically reduced by miR-411-3p mimics but increased by the miR-411-3p inhibitor. However, there were no significant changes in the KIAA0087-MUT groups (Fig. 4d). Additionally, the RIP assay confirmed the direct interaction of miR-411-3p with KIAA0087 (Fig. 4e). FISH assays further demonstrated the colocalization of KIAA0087 and miR-411-3p in the cytoplasm of OS cells (Fig. 4f). Thus, KIAA0087 could bind to miR-411-3p and negatively regulate its expression in OS.

### Upregulation of miR-411-3p confers OS cell growth and proliferation

**Fig. 5:**
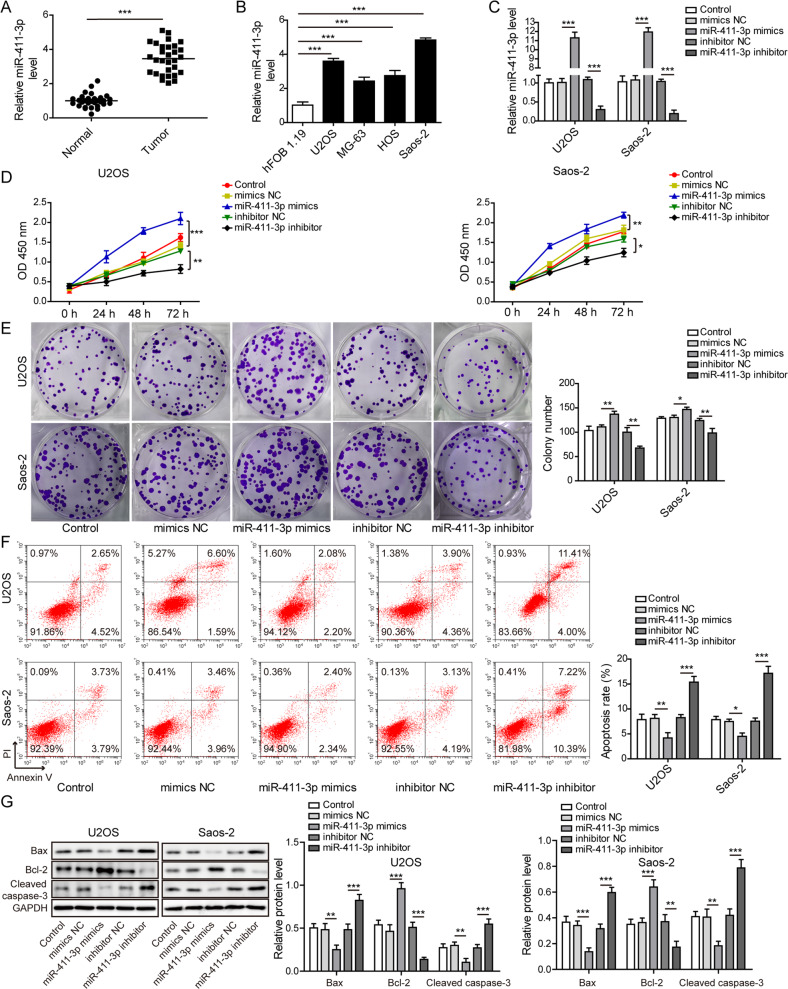
Upregulation of miR-411-3p confers OS cell malignant growth. **a** RT-qPCR analysis was used to evaluate miR-411-3p levels in clinically normal and OS samples (*n* = 30). **b** RT-qPCR analysis was used to evaluate miR-411-3p levels in hFOB 1.19 and different OS cells (U2OS, Saos-2, MG-63, and HOS). **c** RT-qPCR assay was used to validate lentivirus-mediated overexpression or inhibition of miR-411-3p in OS cells. **d** CCK-8 assay determined the proliferation of OS cells. **e** Colony formation assay for evaluating the growth of OS cells. **f** Flow cytometry for testing the apoptotic rate of OS cells. **g** Western blot analysis of the protein levels of Bax, bcl-2, and cleaved caspase-3 in OS cells. *p < 0.05, **p < 0.01, and ***p < 0.001.

Since KIAA0087 could sponge miR-411-3p and repress miR-411 expression in OS cells, we further focused on the expression and functional roles of miR-411-3p in OS. We observed that miR-411-3p was evidently upregulated in OS tissues (Fig. 5a) and a panel of OS cells (Fig. 5b). Next, the transfection efficiency of miR-411-3p in OS cells was verified by RT-qPCR (Fig. 5c). Functional experiments showed that the miR-411-3p inhibitor restricted growth and proliferation and promoted apoptosis of OS cells. In contrast, miR-411-3p overexpression promoted growth and proliferation and inhibited apoptosis of OS cells (Fig. 5d-f). In addition, the protein levels of Bax and cleaved caspase-3 were reduced, and the bcl-2 level was increased by miR-411-3p mimics, whereas the opposite results were found with the miR-411-3p inhibitor (Fig. 5g). The above observations indicated that miR-411-3p was highly expressed in OS, which contributed to OS cell growth and apoptosis inhibition.

### MiR-411-3p promotes migration, invasion, EMT, and JAK2/STAT3 pathway activation in OS cells

**Fig. 6:**
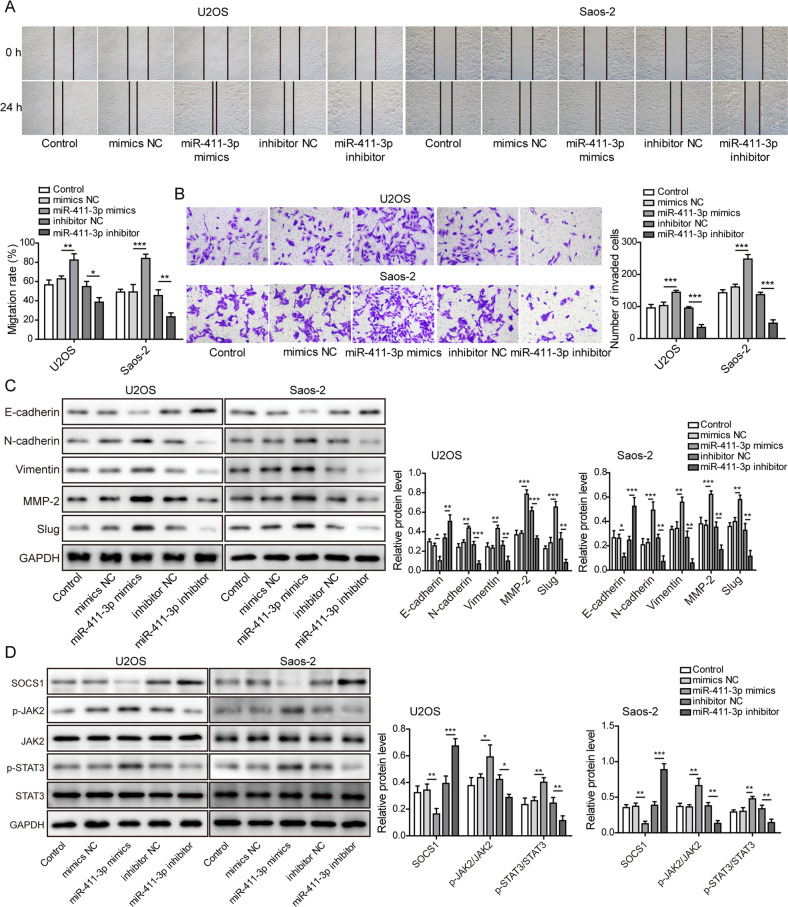
MiR-411-3p overexpression promotes OS cell migration, invasion, and EMT. **a** Wound healing assay for detecting migration in OS cells. **b** Transwell assay for assessing the invasion of OS cells. **c** Western blotting for assessing the levels of EMT-related proteins, including E-cadherin, N-cadherin, vimentin, MMP-2, and slug. **d** Western blot analysis of the expression of SOCS1 and JAK2/STAT3 pathway component proteins. *p < 0.05, **p < 0.01, and ***p < 0.001.

As shown in Fig. 6a, b, the migration and invasion of OS cells were repressed by the miR-411-3p inhibitor but promoted by the miR-411-3p mimic. In addition, the miR-411-3p inhibitor inhibited EMT, as evidenced by the upregulation of E-cadherin and downregulation of N-cadherin, vimentin, MMP-2, and slug. However, these changes were reversed in the miR-411-3p mimic group (Fig. 6c). The p-JAK2 and p-STAT3 protein levels in OS cells were reduced by the miR-411-3p inhibitor but enhanced by the miR-411-3p mimic (Fig. 6d). In addition, transfection with miR-411-3p mimics downregulated SOCS1 protein abundance, whereas miR-411-3p inhibitor transfection upregulated SOCS1 protein abundance (Fig. 6d). These data demonstrated the promotive effects of miR-411-3p on OS cell metastasis in vitro.

### Overexpression of miR-411-3p reverses the phenotypes of KIAA0087-overexpressing OS cells

Next, we evaluated whether miR-411-3p participated in the anticancer effects of KIAA0087 overexpression on OS cells. As determined by RT-qPCR, the decreased expression of miR-411-3p in KIAA0087-overexpressing OS cells was effectively reversed by miR-411-3p mimics (Supplementary Fig. 2a). Importantly, miR-411-3p mimics remarkably abolished the effects of KIAA0087 overexpression on proliferation (Supplementary Fig. 2b, c), apoptosis (Supplementary Fig. 2d), migration (Supplementary Fig. 3a), and invasion of OS cells (Supplementary Fig. 3b). In addition, the weakened EMT mediated by KIAA0087 overexpression was restored by miR-411-3p overexpression (Supplementary Fig. 3c). As shown in Supplementary Fig. 3d, the levels of phosphorylated JAK2 and STAT3 were significantly restricted by KIAA0087 overexpression but restored by miR-411-3p mimics. Additionally, the increased SOCS1 protein induced by KIAA0087 was reversed by miR-411-3p mimics (Supplementary Fig. 3d). Therefore, miR-411-3p partially counteracted the antitumor effects induced by KIAA0087 overexpression.

### MiR-411-3p negatively regulates SOCS1 expression in OS cells

**Fig. 7:**
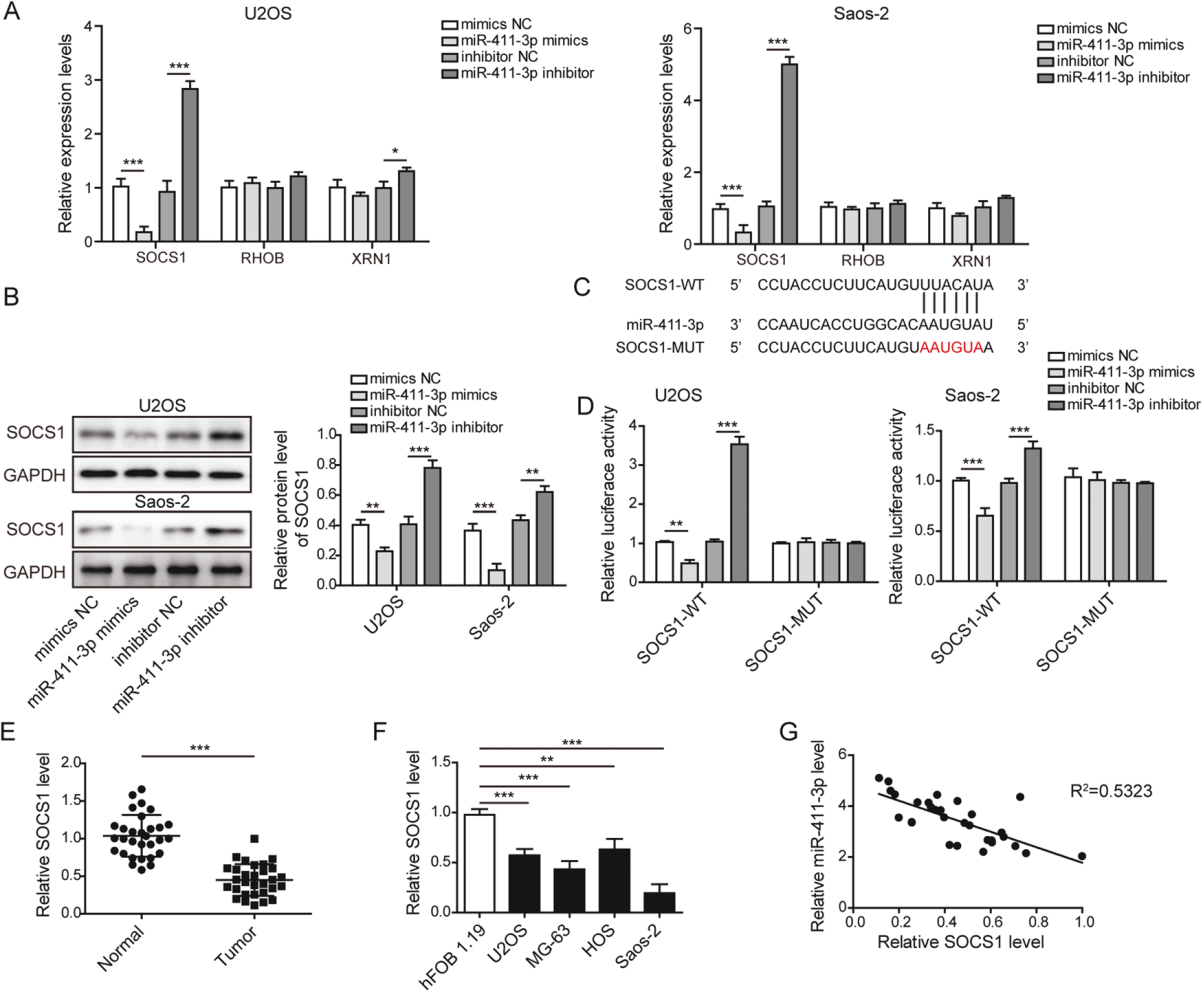
SOCS1 is a target gene of miR-411-3p in OS cells. **a** The mRNA levels of SOCS1, RHOB, and XRN1 were detected by RT-qPCR after overexpression or inhibition of miR-411-3p in OS cells. **b** Western blotting was used to evaluate the protein level of SOCS1 in miR-411-3p mimic- or inhibitor-transfected OS cells. **c** Bioinformatic analysis of the potential miR-411-3p binding sites within the SOCS1 3′-UTR. **d** The direct binding of miR-411-3p to SOCS1 was confirmed by dual-luciferase reporter assay. **e** The expression level of SOCS1 in clinically normal and OS samples (*n* = 30) was determined by RT-qPCR. **f** RT-qPCR analysis of SOCS1 mRNA levels in various OS cells (U2OS, Saos-2, MG-63, and HOS). **g** Spearman correlation analysis of the correlation between the expression of miR-411-3p and SOCS1 in clinical OS samples (*n* = 30). *p < 0.05, **p < 0.01, and ***p < 0.001.

To further investigate the target gene of miR-411-3p, we preliminarily selected three candidates (SOCS1, RHOB, and XRN1) according to prediction ranking and gene function. Further experiments showed that the change in SOCS1 level was most obvious after overexpression or knockdown of miR-411-3p in OS cells (Fig. 7a). As presented in Fig. 7b, the protein level of SOCS1 was strikingly decreased in miR-411-3p-overexpressing cells but increased in miR-411-3p-depleted cells. Furthermore, the putative binding sites between SOCS1 and miR-411-3p were predicted by the StarBase database (Fig. 7c). Moreover, miR-411-3p mimics repressed and miR-411-3p inhibitor enhanced the luciferase activity of SOCS1-WT but not SOCS1-MUT (Fig. 7d). Downregulation of SOCS1 in OS tumor tissues were validated by RT-qPCR assay (Fig. 7e). Consistently, SOCS1 was downregulated in various OS cells (Fig. 7f). A negative correlation between SOCS1 and miR-411-3p in OS tissues was also observed (Fig. 7g). These data revealed that miR-411-3p suppressed SOCS1 expression by binding to its 3′-UTR in OS cells.

### Depletion of SOCS1 counteracts the anticancer effects of the miR-411-3p inhibitor on OS cells

Next, we explored whether miR-411-3p affected the malignant properties of OS cells by regulating SOCS1. RT-qPCR showed that depletion of SOCS1 evidently reversed the miR-411-3p inhibitor-induced promotion of SOCS1 mRNA in OS cells, while it did not affect miR-411-3p levels (Supplementary Fig. 4a, b). Additionally, the increased protein level of SOCS1 induced by the miR-411-3p inhibitor was also abolished by SOCS1 inhibition (Supplementary Fig. 4c). Moreover, the miR-411-3p inhibitor weakened the proliferative, migratory, and invasive abilities but enhanced the apoptosis of OS cells, which was counteracted by silencing SOCS1 (Supplementary Fig. 4d–f and Supplementary Fig. 5a, b). Additionally, depletion of SOCS1 significantly restricted miR-411-3p inhibitor-mediated EMT inhibition in OS cells (Supplementary Fig. 5c). The reduced p-JAK2 and p-STAT3 levels in miR-411-3p inhibitor-transfected OS cells were partly restored by SOCS1 knockdown (Supplementary Fig. 5d). Collectively, miR-411-3p regulated the malignant phenotypes of OS cells through SOCS1.

### KIAA0087 overexpression or miR-411-3p knockdown represses the growth and lung metastasis of OS cells in vivo

**Fig. 8:**
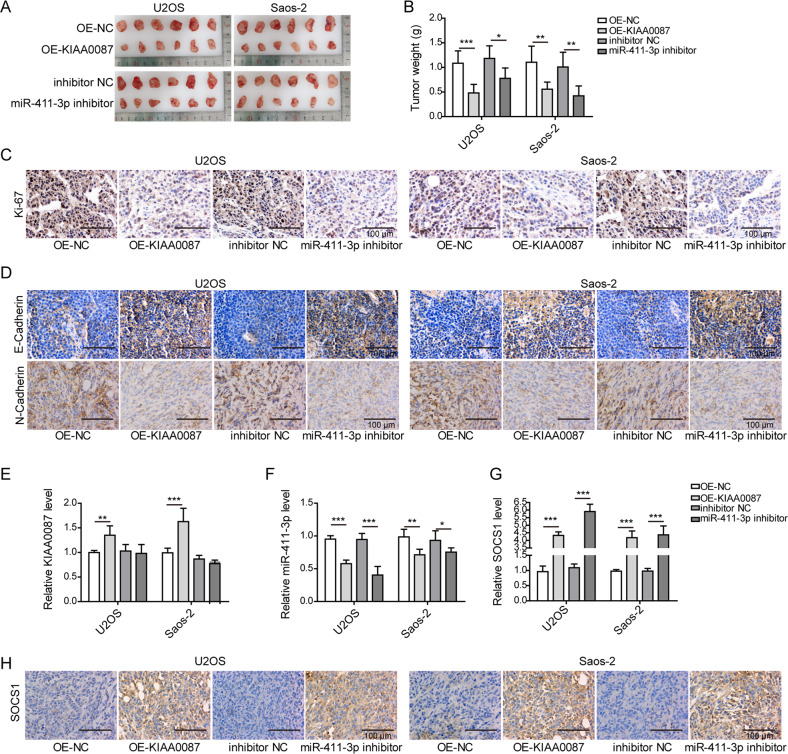
KIAA0087 overexpression or miR-411-3p knockdown disrupts tumor growth in vivo. **a**, **b** Xenograft tumors (**a**) and quantitative results (**b**) of tumor weight from various groups. **c** Ki-67 expression in tumor tissues was determined by immunohistochemical staining. **d** E-cadherin and N-cadherin expression in tumor tissues was determined by immunohistochemical staining. **e**–**g** RT-qPCR for determining KIAA0087 (**e**), miR-411-3p (**f**), and SOCS1 (**g**) expression in tumor tissues. **h** SOCS1 expression in tumor tissues was detected by immunohistochemical staining. *p < 0.05, **p < 0.01, and ***p < 0.001.

**Fig. 9:**
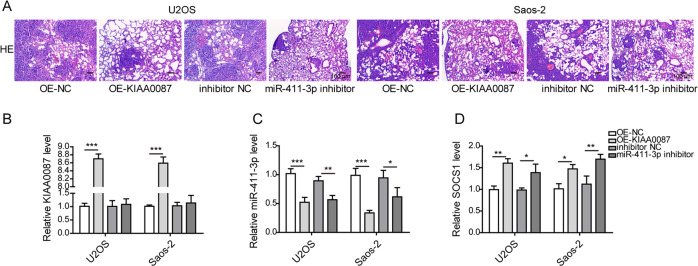
KIAA0087 overexpression or miR-411-3p inhibition suppresses lung metastasis in vivo. **a** HE staining for evaluating metastatic tumor cells in lung tissues. **b**–**d** RT-qPCR for detecting KIAA0087 (**b**), miR-411-3p (**c**), and SOCS1 (**d**) expression in the lung tissues of nude mice. *p < 0.05, **p < 0.01, and ***p < 0.001.

Finally, we assessed the roles of KIAA0087 or miR-411-3p in the carcinogenesis of OS in vivo. To achieve this, U2OS and Saos-2 cells stably infected with lentiviruses carrying KIAA0087 or miR-411-3p inhibitor were injected into nude mice. Obvious decreases in tumor weight and volume in the KIAA0087-overexpression or miR-411-3p-inhibition group were demonstrated (Fig. 8a, b). In addition, a lower Ki-67 expression level in tumor tissues was confirmed in the KIAA0087-overexpression or miR-411-3p-inhibition group (Fig. 8c). Overexpression of KIAA0087 or inhibition of miR-411-3p increased the E-cadherin level but suppressed the N-cadherin expression level in tumor tissues, suggesting that the EMT process was repressed (Fig. 8d). In addition, KIAA0087 overexpression led to a decreased miR-411-3p expression level and an increased SOCS1 expression level in tumor tissues (Fig. 8e–h). Additionally, SOCS1 was upregulated in the miR-411-3p-inhibition group (Fig. 8g, h). Moreover, the in vivo metastatic capacity of OS cells was also assessed. As presented in Fig. 9a, H&E staining indicated that KIAA0087 overexpression or miR-411-3p inhibition strikingly reduced the area of metastatic OS cells in the lung tissues (Fig. 9a). The expression level of miR-411-3p declined, while SOCS1 expression was enhanced by KIAA0087 overexpression in the lung tissues (Fig. 9b–d). Accordingly, the expression of SOCS1 was enhanced in the lung tissues of the miR-411-3p-inhibition group (Fig. 9c, d). Collectively, the in vivo growth and lung metastasis of OS cells were suppressed by KIAA0087 overexpression or miR-411-3p inhibition.

## Discussion

Although improvement has been made in the 5-year survival rate of OS patients in recent years, recurrent or metastatic OS is still refractory to cancer treatments^19^. Therefore, identifying more effective therapies for treating refractory OS is urgently needed. Here, we uncovered the biological functions of a novel lncRNA, KIAA0087, in the progression of OS. We found that KIAA0087 was downregulated, but miR-411-3p was upregulated in OS tissues and cells, which facilitated the growth, metastasis, and EMT of OS cells in vitro and in vivo. Mechanistically, KIAA0087 sponged miR-411-3p to enhance SOCS1-mediated inactivation of the JAK2/STAT3 pathway. Our data demonstrated the potential value of KIAA0087 and miR-411-3p as effective targets for treating refractory OS.

In recent years, the biological functions of lncRNAs in cancers have attracted wide attention. Growing evidence has indicated that many lncRNAs are dysregulated in OS, which contributes to the malignant development of OS. For example, TUG1 is overexpressed in OS, and TUG1 silencing inhibits the proliferation and invasion of OS cells^20^. Han et al. showed that lncRNA ATB facilitates OS cell growth and metastasis and can serve as a therapeutic target^21^. In our study, we focused on a novel lncRNA, KIAA0087. To date, limited studies have investigated the functions of KIAA0087 in OS. For the first time, we revealed that KIAA0087 was downregulated in OS tissues and cells, which was positively correlated with the TNM stage of OS patients. However, KIAA0087 expression was not significantly correlated with distant metastasis in OS patients, which might be caused by our limited sample size and individual and geographical differences in patients. EMT is a key process in the metastasis and progression of OS and plays pivotal roles in chemotherapy resistance, recurrence, and poor prognosis^22,23^. EMT induction is responsible for malignant cell metastasis during OS progression^24^. Therefore, inhibiting the progression of EMT may be a potentially effective treatment for OS. In this study, we aimed to explore the underlying mechanism of EMT and metastasis in OS. Thus, a series of EMT-related signaling pathways that might be regulated by KIAA0087 were screened. We found that the JAK2/STAT3 pathway could be obviously affected by KIAA0087 in OS cells. Moreover, upregulation of KIAA0087 repressed OS cell growth, metastasis, and EMT and triggered apoptosis in vitro and in vivo. These observations suggest that KIAA0087 is a tumor suppressor in OS.

We further explored the underlying mechanisms by which KIAA0087 regulates the development of OS. By bioinformatics analysis, five target miRNAs (miR-30e-5p, miR-135b-5p, miR-6807-3p, miR-488-3p, and miR-411-3p) of KIAA0087 were screened preliminarily. Further results showed that the change in miR-411-3p level was apparently most pronounced after regulation by KIAA0087 in OS cells. Thus, miR-411-3p was a focus of this study. In previous studies, miR-411-3p has been reported to affect tumor development. For instance, miR-411-3p, as a target of lncRNA TTN-AS1, is involved in the pathogenesis of oral squamous cell carcinoma^25^. Here, we provided the first evidence that miR-411-3p upregulation promoted the malignant properties of OS cells. More importantly, we found that miR-411-3p was elevated by KIAA0087 knockdown but reduced by KIAA0087 overexpression. In addition, the direct interaction between miR-411-3p and KIAA0087 was validated by dual-luciferase, RIP, and FISH assays. Further rescue experiments demonstrated that miR-411-3p mimics effectively reversed the antitumor effects of KIAA0087 overexpression on OS cells. Therefore, KIAA0087 downregulation promoted tumorigenesis of OS via direct upregulation of miR-411-3p expression. Our research team reported for the first time that KIAA0087 could sponge miR-411-3p.

We further selected SOCS1 as the target gene of miR-411-3p because the miR-411-3p-mediated level change in SOCS1 was the most pronounced. SOCS1 is downregulated in OS tissues and cells^18^. SOCS1 has been shown to repress EMT-mediated metastasis and invasion in cervical cancer^26^. David et al reported that SOCS1 suppressed the metastatic progression of colorectal cancer by reversing EMT^27^. Consistent with these studies, we found a reduction in SOCS1 expression in OS tissues and cells, which negatively correlated with miR-411-3p expression in OS patients. More importantly, SOCS1 expression was enhanced by a miR-411-3p inhibitor but reduced by miR-411-3p mimics. The direct binding between miR-411-3p and SOCS1 was confirmed by dual-luciferase assay. Furthermore, silencing SOCS1 reversed the antitumor effects of the miR-411-3p inhibitor on OS cells. Therefore, miR-411-3p affected OS progression by targeting SOCS1. This is the first evidence of the direct link between miR-411-3p and SOCS1 in OS.

SOCS1 is a potent suppressor of the JAK2/STAT3 pathway through proteasomal degradation of JAK2^26^. JAK2 is an important member of the JAK family, which regulates various biological functions, such as proliferation, migration, and apoptosis^28^. The phosphorylation of JAK2 can lead to the activation of STAT3^29^. Mounting evidence has demonstrated that the activation of the JAK2/STAT3 pathway participates in the growth and metastasis of OS^30–32^. STAT3 can promote the EMT process in tumors by enhancing vimentin expression^33^. In the present study, the activation of the JAK2/STAT3 pathway was suppressed by forced expression of KIAA0087 or miR-411-3p inhibitor. Therefore, the JAK2/STAT3 pathway participated in KIAA0087/miR-411-3p/SOCS1 axis-mediated tumorigenesis of OS.

We are aware that this study has several limitations. First, we did not investigate what caused the downregulation of KIAA0087 in OS. Second, the identification of potential EMT-related signaling networks regulated by KIAA0087 using bioinformatics data may expand the pathological mechanisms of OS development. Third, we mainly explored the function and related mechanisms of KIAA0087 in the EMT and metastasis of OS cells. Other biological functions of KIAA0087 in OS remain unclear and need to be explored in future studies. Nevertheless, this research is the first to reveal that KIAA0087 promotes SOCS1-mediated inactivation of the JAK2/STAT3 pathway by sponging miR-411-3p, which suppresses the growth and metastasis of OS cells in vitro and in vivo. Our findings provide new insights into molecular mechanisms of OS oncogenesis and a potential therapeutic target.

## Supplementary information

Supplementary information
