# Supplementary material for: LncRNA KIAA0087 suppresses the progression of osteosarcoma by mediating the SOCS1/JAK2/STAT3 signaling pathway
Source: Exp Mol Med. 2023 Apr 3;55(4):831–43. doi: 10.1038/s12276-023-00972-8 (PMC10167219; doi:10.1038/s12276-023-00972-8)
Supplement: Supplementary file 1 — Supplementary information [file 12276_2023_972_MOESM1_ESM.pdf]

Supplementary figure legends

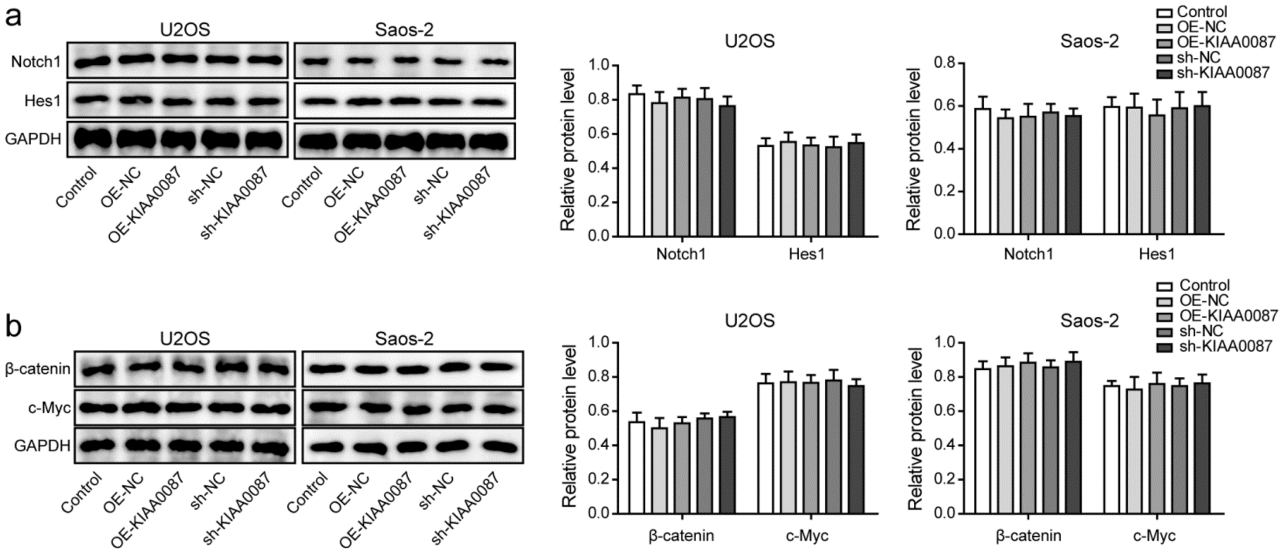

Supplementary Fig. 1 Effect of KIAA0087 on other EMT-related signaling pathways.

(a) Western blot analysis of Notch1/Hes1 pathway component proteins after KIAA0087 overexpression or knockdown. (b) The protein levels of Wnt/β-catenin pathway component proteins were evaluated by western blot.

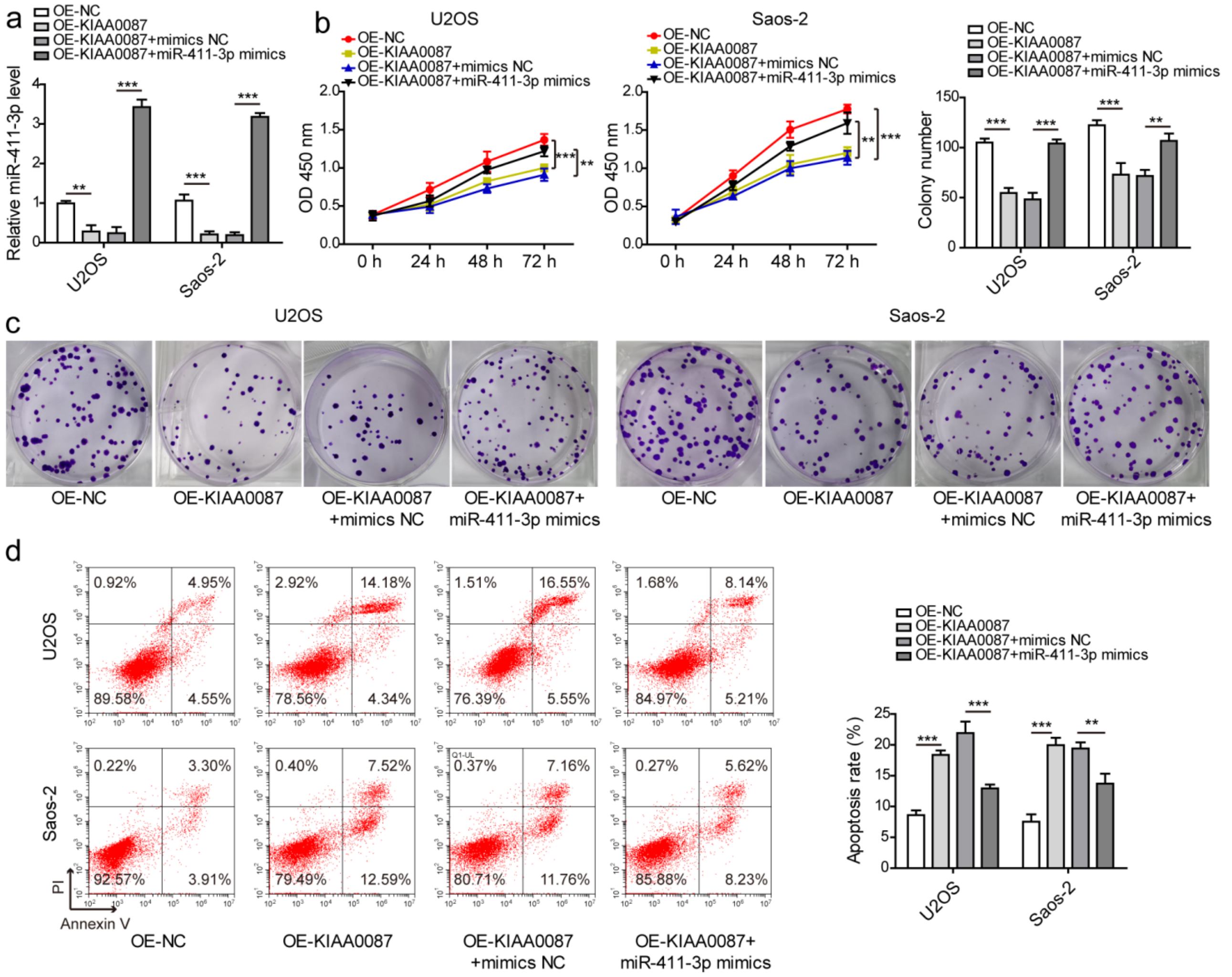

Supplementary Fig. 2 MiR-411-3p is responsible for the anti-tumor roles of KIAA0087 on growth and apoptosis in OS cells.

(a) RT-qPCR for miR-411-3p expression in OS cells after co-overexpression of KIAA0087 and miR-411-3p. (b) CCK-8 assay determined the proliferation of OS cells. (c) Colony formation assay for evaluating the growth of OS cells. (d) Flow cytometry for testing the apoptotic rate of OS cells. ** $p < 0.01$, and *** $p < 0.001$.

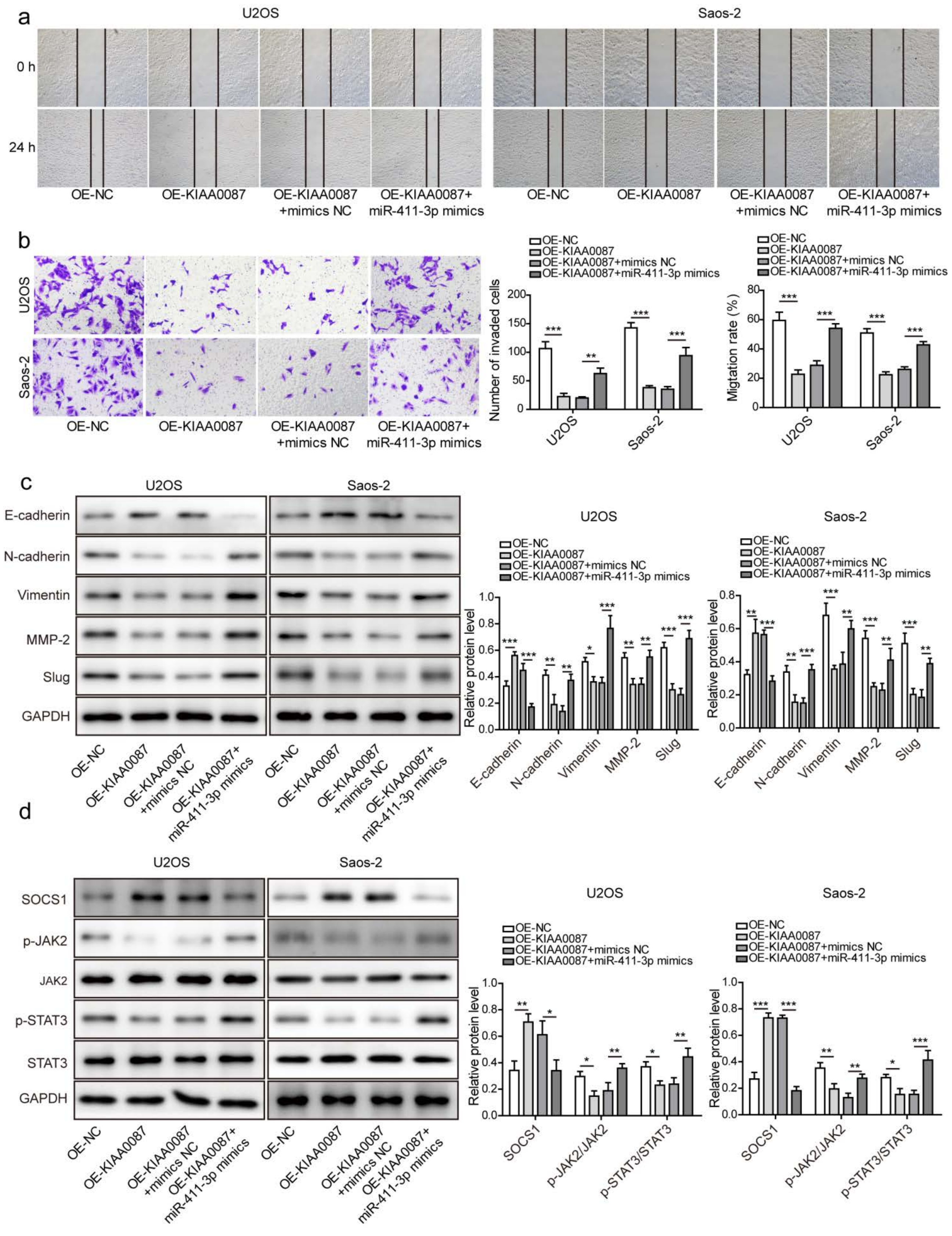

Supplementary Fig. 3 MiR-411-3p is involved in the inhibitory effects of KIAA0087 on migration, invasion, and EMT of OS cells.

(a) Wound healing assay was performed to determine migration of OS cells. (b) Transwell assay was used to assess the invasive ability of OS cells. (c) Western blot of E-cadherin, N-cadherin, vimentin, MMP-2, and slug expression in U2OS and Saos-2 cells. (d) Western blot detected the protein levels of SOCS1, p-JAK2, JAK2, p-STAT3, and STAT3. * $p < 0.05$, ** $p < 0.01$, and *** $p < 0.001$.

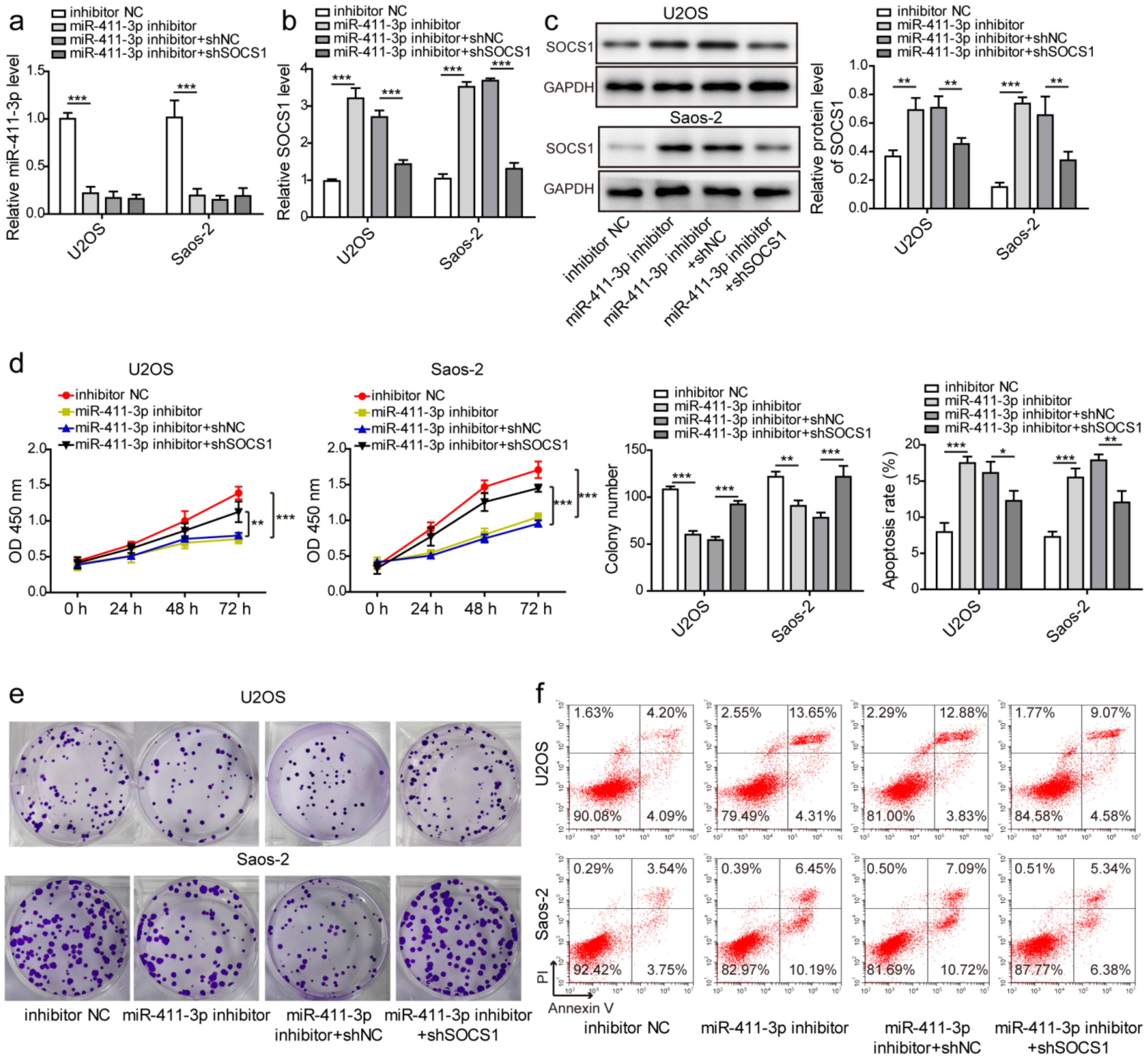

Supplementary Fig. 4 MiR-411-3p inhibitor represses the malignant growth of OS cells via regulating SOCS1.

(a-b) RT-qPCR for miR-411-3p (a) and SOCS1 (b) expression levels in OS cells. (c) SOCS1 protein level in OS cells was assessed by western blot. (d) CCK-8 assay determined the proliferation of OS cells. (e) Colony formation assay for evaluating the growth of OS cells. (f) Flow cytometry for testing the apoptotic rate of OS cells. * $p < 0.05$, ** $p < 0.01$, and *** $p < 0.001$.

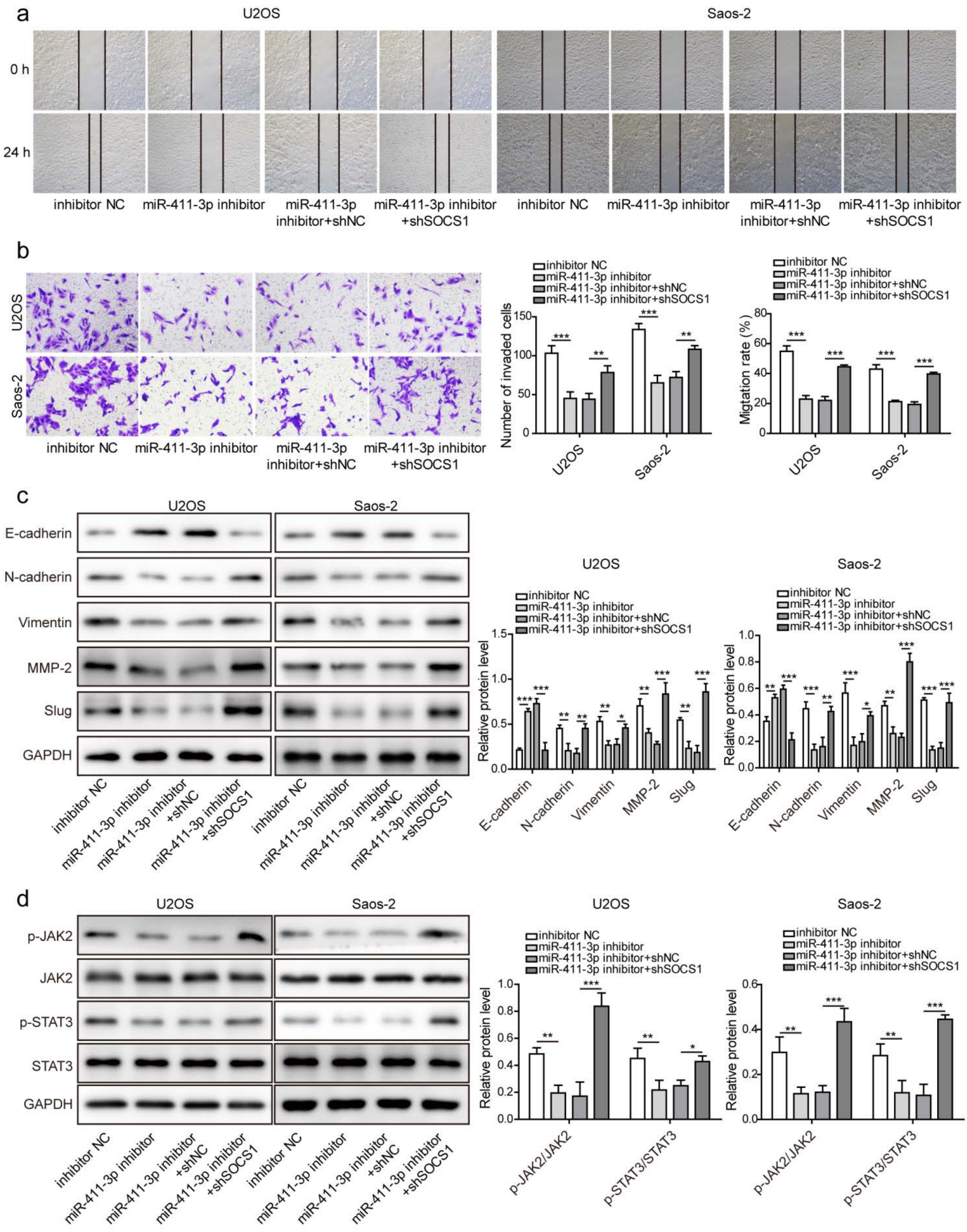

Supplementary Fig. 5 MiR-411-3p inhibitor represses the migration, invasion, and EMT of OS cells via regulating SOCS1.

(a) Wound healing assay was performed to detect the migrative ability of OS cells. (b) Transwell

assay for assessing the invasive ability of OS cells. (c) E-cadherin, N-cadherin, vimentin, MMP-2, and slug expression in U2OS and Saos-2 cells was detected by western blot. (d) JAK2/STAT3 pathway component proteins were detected by western blot. * $p < 0.05$, ** $p < 0.01$, and *** $p < 0.001$.
